# Supplementary material for: Advancing the Indian cattle pangenome: characterizing non-reference sequences in Bos indicus
Source: J Anim Sci Biotechnol. 2025 Feb 7;16:21. doi: 10.1186/s40104-024-01133-1 (PMC11804092; doi:10.1186/s40104-024-01133-1)
Supplement: Supplementary file 2 — Additional file 2: Fig S1. Upset plot for the shared NUIs among five Bos indicus breeds from NUI discovery pipeline. Fig S2. Upset plot for the shared NUIs among five Bos indicus breeds from minigraph pipeline. Fig S3. Mapping rate of transcriptome sequencing reads to Brahman reference and pangenome. Fig S4. Volcano plot of differentially expressed NUI genes. Fig S5. Cladogram of 98 samples using presence and absence of NUIs. Fig S6. GO annotation of BICIs. [file 40104_2024_1133_MOESM2_ESM.docx]

**
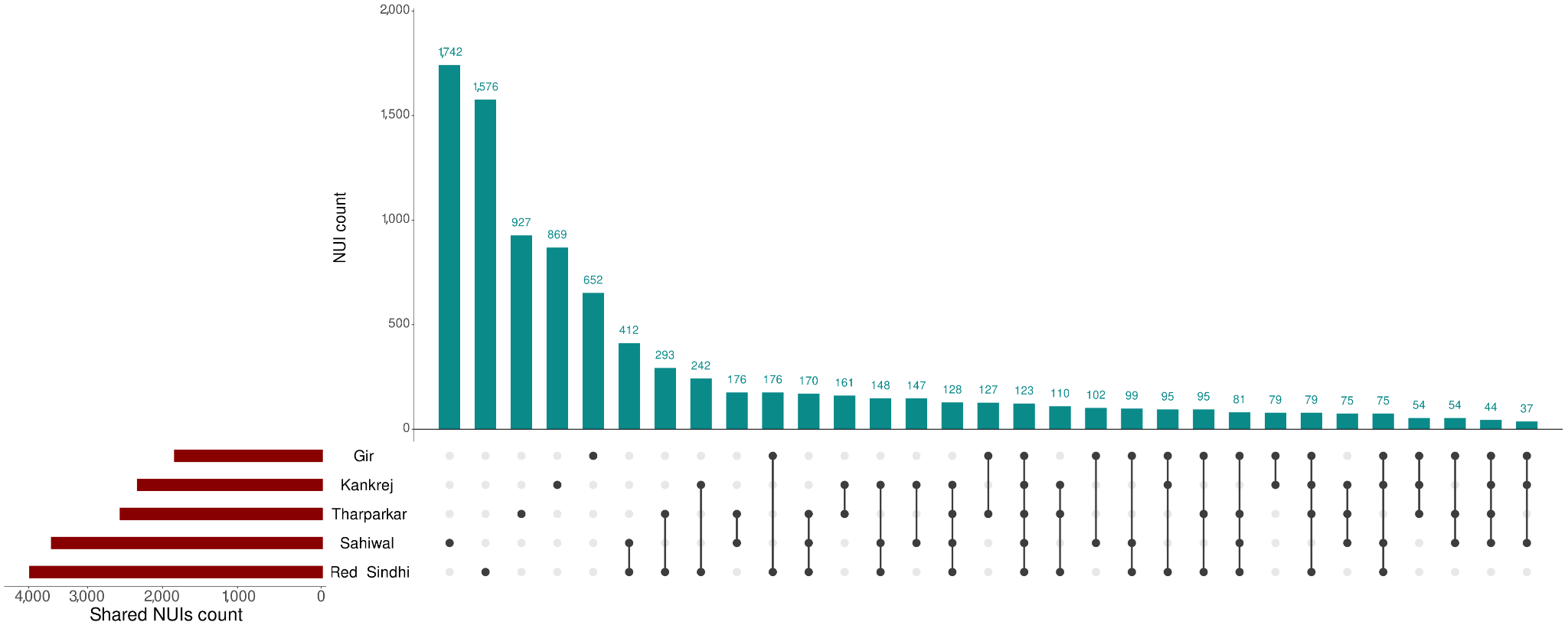
**

**Fig S1: Upset plot for the shared NUIs among five *Bos indicus* breeds from NUI discovery pipeline**. Each vertical bar in the chart signifies the number of NUIs shared by the corresponding combination of breeds listed below. The horizontal bar chart represents the count of these shared NUIs.

**
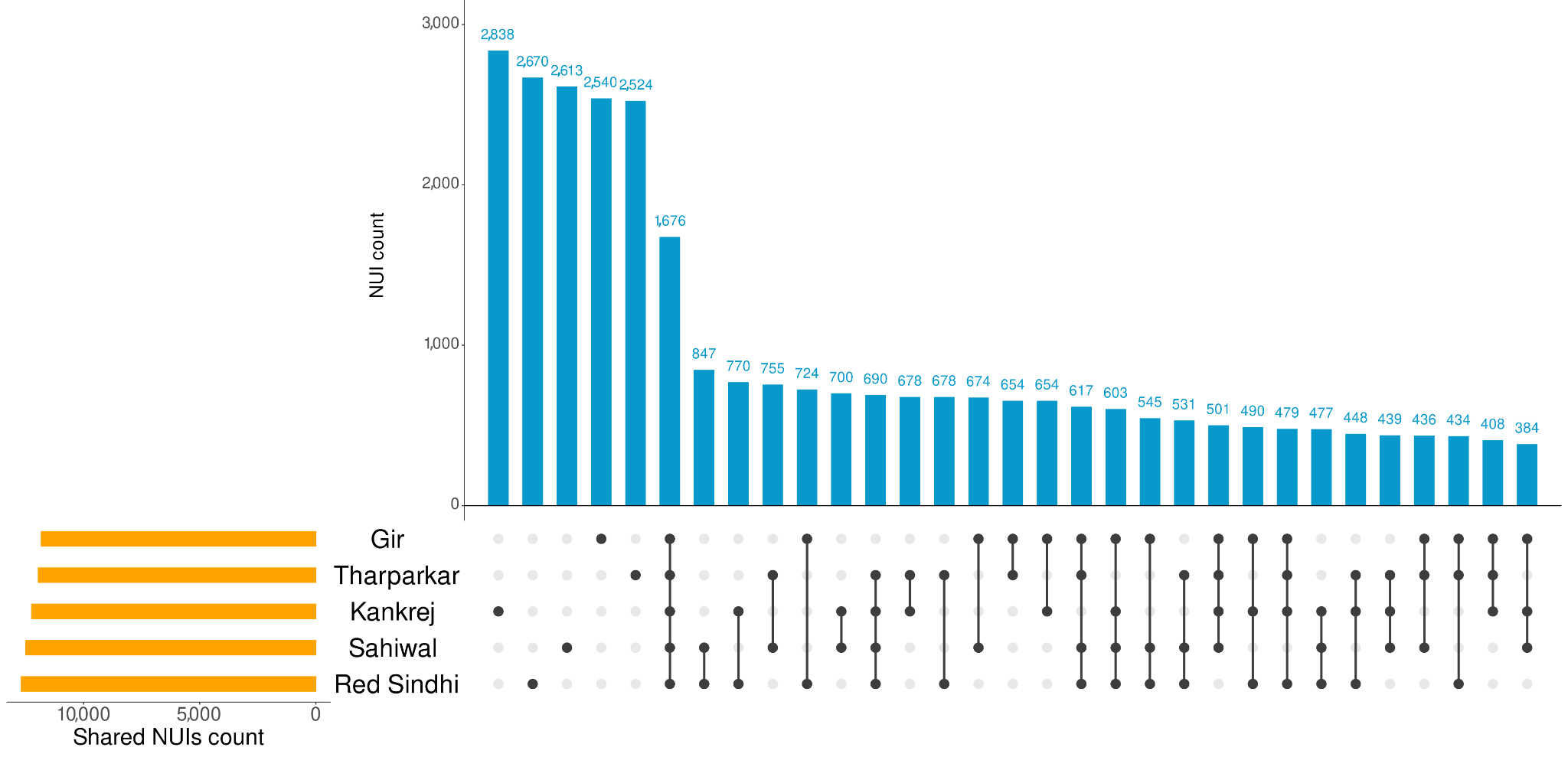
**

**Fig S2: Upset plot for the shared NUIs among five *Bos indicus* breeds from minigraph pipeline.** Each vertical bar within the chart denotes the quantity of NUIs shared by the respective combination of listed breeds below. The horizontal bar graph indicates the tally of these shared NUIs.

**
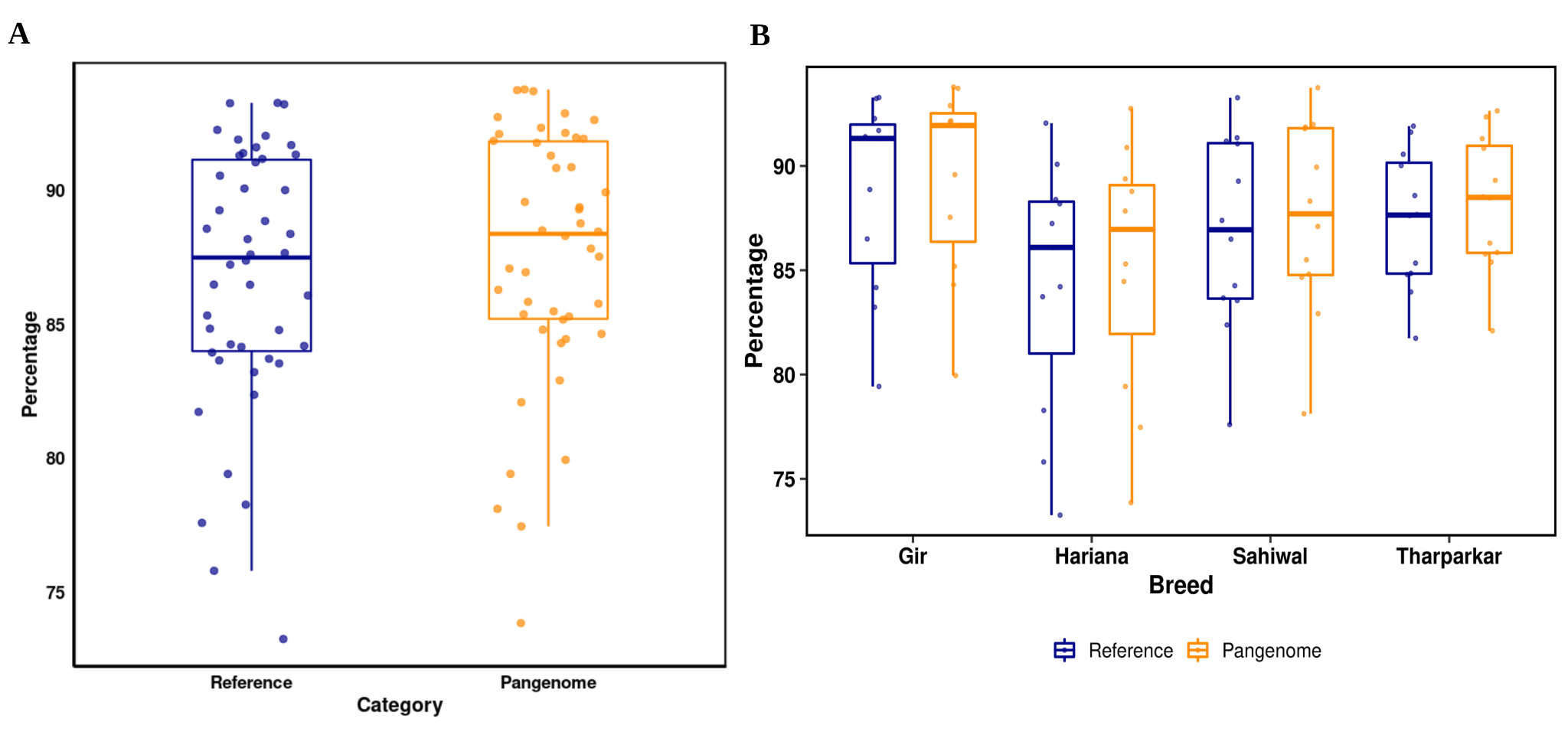
**

**Fig S3: Mapping rate of transcriptome sequencing reads to Brahman reference and pangenome. (A)** The boxplot displays the percentage of transcriptome reads mapped to the Brahman reference genome and the pangenome. The Y-axis represents the mapping percentage, with the pangenome showing a higher mapping rate compared to the Brahman reference. **(B)** A pairwise boxplot compares the mapping percentages of transcriptome reads from four *desi* cattle breeds against both the Brahman reference genome and the pangenome.

**
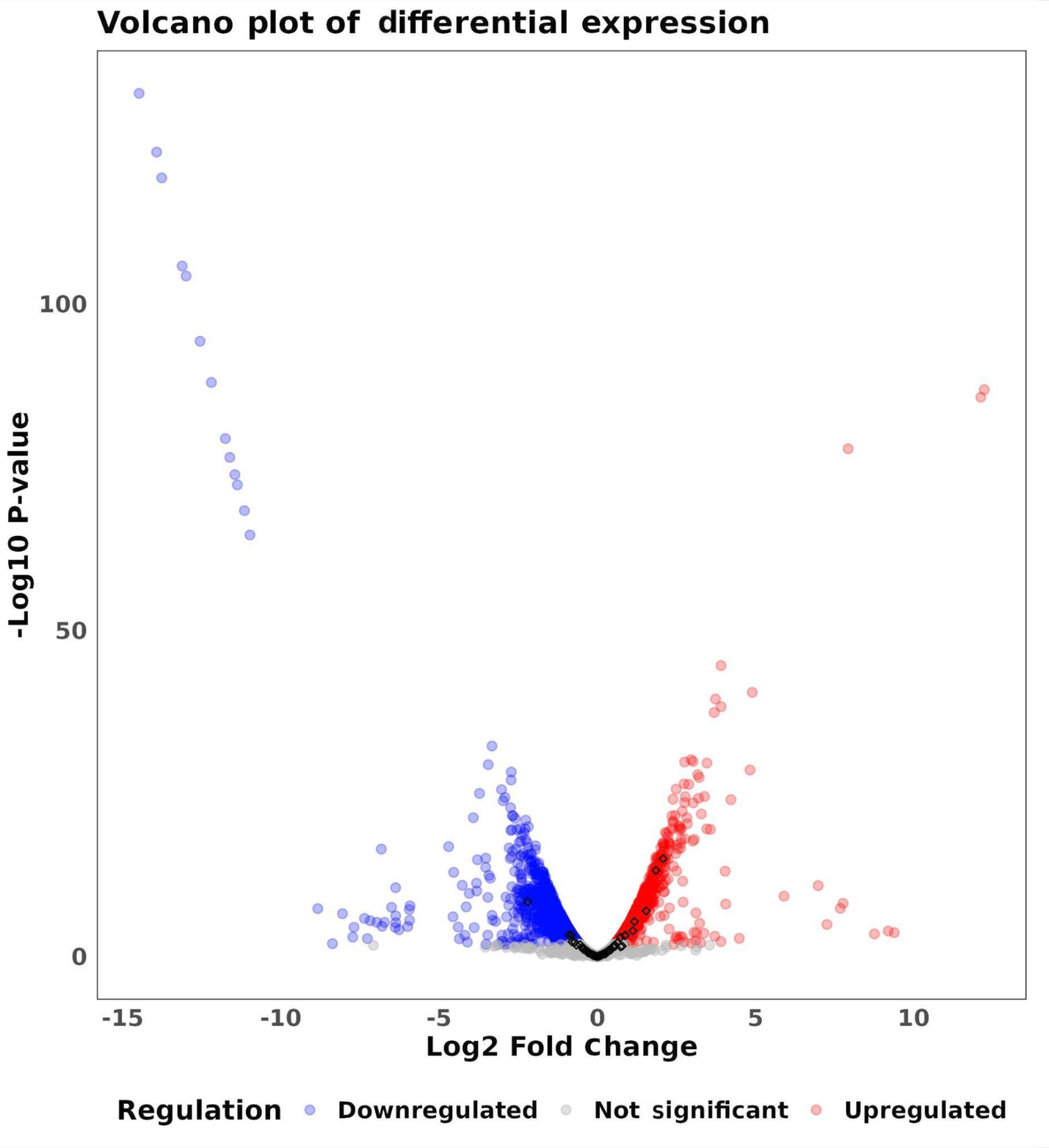
**

**Fig S4: Volcano plot of differentially expressed NUI genes.** Volcano plot illustrates the results of a differential expression analysis. Genes significantly upregulated and downregulated (FDR ≤ 0.05) in response to heat stress in Hariana breed of *desi* cattle are shown in red and blue, respectively. The small black diamond markers highlight the 84 genes identified from non-reference genomic sequences.


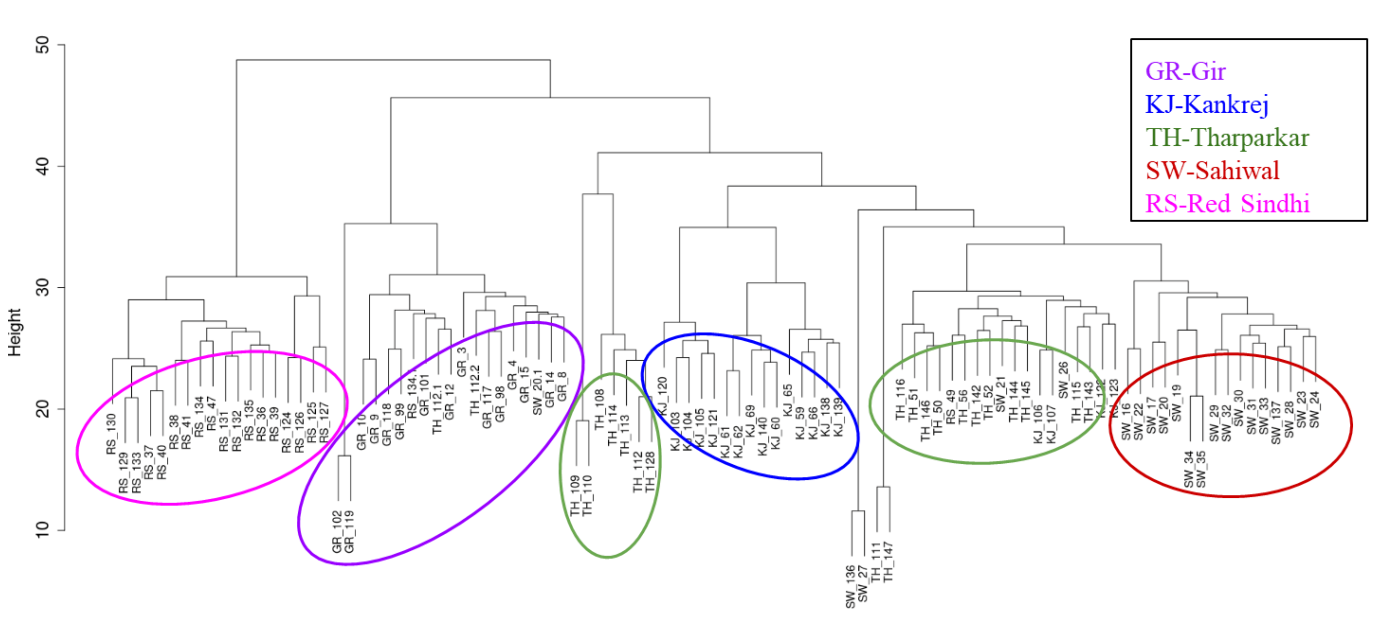


**Fig S5: Cladogram of 98 samples using presence and absence of NUIs.** The breeds are clustered based on Euclidean distance of the presence or absence of NUIs.

**
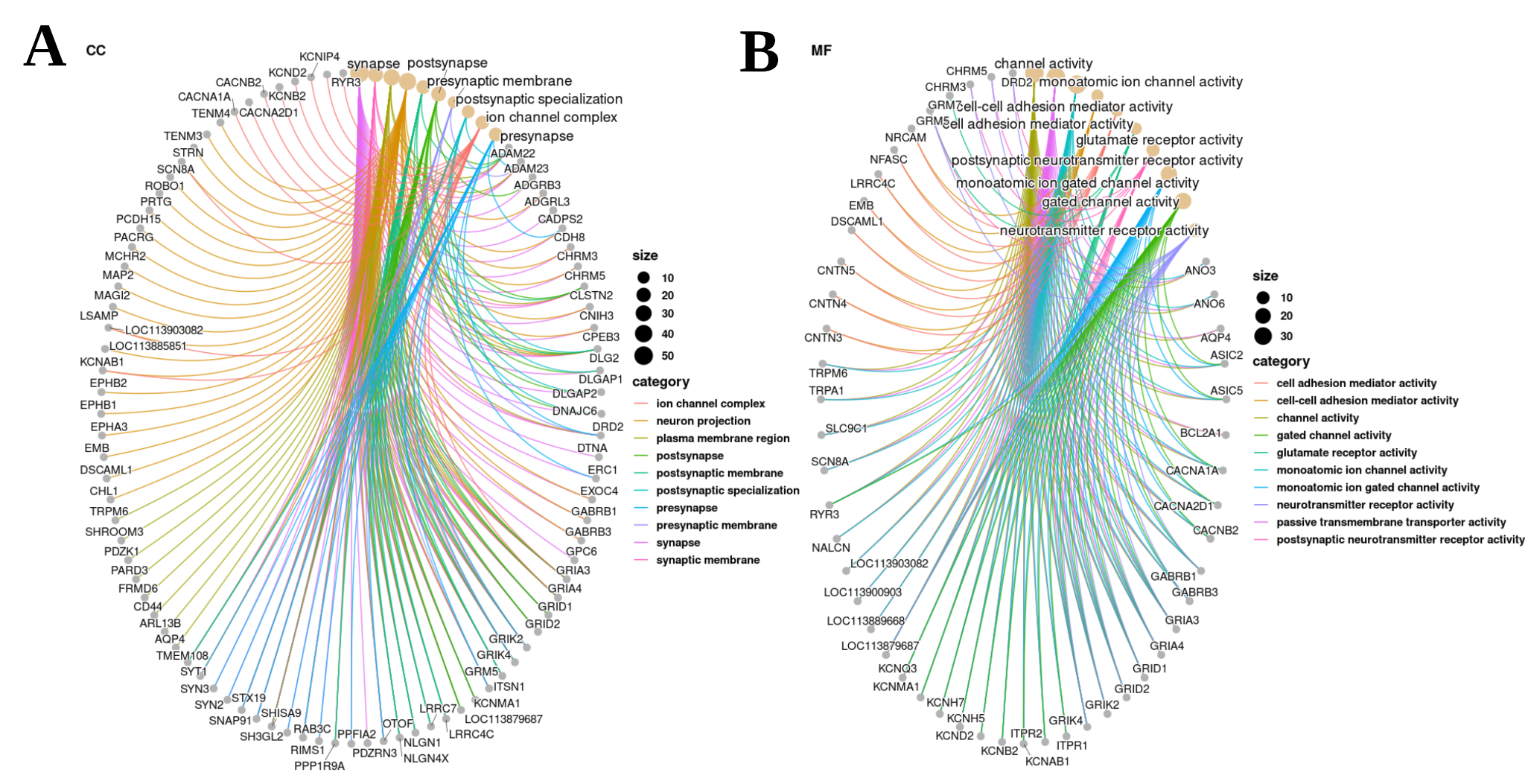
**

**Fig S6: GO annotation of BICIs. (A)** The Cnet plot displays the enriched terms and genes associated with cellular components (CC). **(B)** The Cnet plot illustrates the enriched terms and genes pertaining to molecular function (MF).
